# Riding the hilltop: practical implementation and assessment of an implicit hilltop illusion

**DOI:** 10.3389/fneur.2025.1623749

**Published:** 2025-08-07

**Authors:** Carole-Anne Vollette, Christopher J. Bockisch, Giovanni Bertolini

**Affiliations:** ^1^University of Zürich, Zürich, Switzerland; ^2^Departments of Ophthalmology, Neurology and Otorhinolaryngology, Head and Neck Surgery, University Hospital Zürich, University of Zürich, Zürich, Switzerland; ^3^University of Applied Sciences and Arts Northwestern Switzerland, Windisch, Switzerland

**Keywords:** vestibular system, sensory, illusion, perception, otolith, gravity

## Abstract

Passive motions can lead to conflicting combinations of visual and vestibular signals that can have a tremendous impact on our ability to navigate and comprehend the world. However, conflicting motion signals are also exploited for rehabilitation, adaptation training, and entertainment by creating functional illusions (VR, amusement parks). Low-frequency linear translations can induce “hilltop illusions,” a perceptual phenomenon consisting in a reinterpretation of the inertial acceleration as tilt with respect to gravity. Compared to other vestibular stimuli, the hilltop illusion has rarely been used as it is considered unpractical due to the complexity of the necessary motion devices, which can have high operational costs, induce cognitive biases jeopardizing the illusion and discomfort in the subject. We hypothesize that a practical protocol to create and quantify a hilltop illusion can be realized using low-frequency (0.16 Hz) small-amplitude (0.45 m) translations on a standard motion simulator (Stewart Platform), provided that expectations and awareness of the illusion are hindered. To this aim, we combined the lateral oscillations with 90° phase-shifted roll movement with a random direction and amplitude. A consistent tilt illusion was measured across 12 healthy participants (29.7 ± 14.5 yo, 6 females). The hilltop illusion was quantified using both haptic vertical (HV) and subjective visual vertical (SVV) assessments, showing a significant tilt perception with larger values displayed in HV (2.2° ± 1.2—gain to the GIA tilt = 41.8%) compared to SVV (0.51° ± 0.57—gain = 9.7%). The protocol was well tolerated, with minimal motion sickness reported. This new protocol offers an accessible method for the generation of an implicit vestibular illusion, while demonstrating the importance of preventing cognitive awareness of motion cues. It offers insights in the perceptual process of vestibular conflicts and provides a foundation for potential development of diagnostic and therapeutic applications, for training and for illusions.

## Introduction

Visual, vestibular and proprioceptive inputs are necessary for accurate environmental navigation and perception (1). While the visual system extracts information on self-motion from the observed scene and the proprioceptive system located in our muscles and joints provides a sense of body position awareness, our vestibular system uses two inertial sensors: the semi-circular canals, the biological equivalent of gyroscopes that can sense rotational velocity, and the otoliths organs, biological linear accelerometers that can sense the gravito-inertial acceleration (GIA). Information from different sensory modalities combines into the best estimate of self-motion, accounting for expectations and the reliability of each sensor. When visual information is uncertain or conflicts with what is expected, the vestibular system plays a critical role in resolving such discrepancies (2, 3) and vice-versa. Knowing the functions and limitations of each sensory modality and their integration process assists in the understanding of when and how interpretation error occurs, leading to sensory conflicts, physiological self-motion illusions or perceptual disorders. This is also of practical importance as it can help identify and predict situations where sensory conflicts occur, shaping the development of emerging technology such as extended reality or self-driving cars, and it can suggest new approaches to diagnose vestibular perceptual disorders. Notably, sensory conflicts and uncertainty can also be exploited to drive adaptation or rehabilitation (4–6).

A cardinal challenge in the generation of a stable percept of self-motion and self-orientation is the inherent ambiguity between tilt and translation as sensed by any accelerometer (7, 8). Since gravitational and inertial linear accelerations cannot be distinguished by accelerometers (i.e., the otoliths), a head tilt with respect to gravity cannot be distinguished from a linear acceleration in the opposite direction. Two hypotheses have been proposed to explain how the brain may resolve this ambiguity. According to the frequency segregation hypothesis, high frequency GIA tilts are interpreted as translation, while low frequencies GIA tilts are interpreted as tilts (9–11). Alternatively, the multisensory hypothesis proposes that the brain uses information from a gyroscope (i.e., semi-circular canals) to infer an orientation with respect to gravity and separate it from the inertial acceleration (2, 12). The two hypotheses have some functional overlap. As semi-circular canals are high pass filters of head velocity, the ambiguity in sensed angular motion at low frequency leads to errors in the estimate of gravity direction (13). Consequently, the multisensory integration hypothesis also predicts a frequency dependent effect. Studies have investigated the tilt-translation ambiguity in different conditions (14), bringing considerable advancements in our understanding on self-motion perception, demonstrating neural correlates of models and shedding light on spatial disorientation in patients (15, 16) and workers exposed to unnatural motion stimuli (aircraft pilots, astronauts) (17). A complete resolution of the tilt-translation ambiguity is not performed by the brain, however. Horizontal eye movement compatible with leftward translation occurs during static head tilt, as if part of the gravitational acceleration would be interpreted as inertial acceleration (18). The need to resolve GIA in gravitational and inertial acceleration is not all-encompassing and critically depends on the conditions. For example, maintaining postural stability may benefit from counteracting the GIA (no need to separate gravity from inertial acceleration), while the observation of the environment may require defining a gravitational reference frame. The tilt-translation ambiguity has also been widely studied with respect to the different perceptual illusions it induces (19, 20).

Despite this large effort, little has been done to explore the potential of artificial situations where the necessary resolution of tilt-translation ambiguity challenges the brain (i.e., perceptual illusion of verticality) as a basis for adaptation and rehabilitation. This contrasts starkly with visual based conflicts, which have been used in recent years for rehabilitation protocols exploiting dynamic (optokinetic, vection) and static (tilted scene) stimuli (21). It also marks a fundamental difference with respect to other forms of vestibular based conflicts (e.g., Cross-coupling), which have been widely used and developed in stepwise paradigms to decrease sensitivity to airsickness (22). This difference may have different roots. Although linear oscillations have been successfully tested in the past as training against motion sickness (23), they are usually considered highly nauseogenic (24) and even though few studies followed up (25), no established training paradigm arose. However, any condition where a sensory conflict is created implies an error signal for the brain that can be exploited to support an adaptation process, provided that the exposure dosage is appropriately controlled (4, 26–28). The difference to other vestibular-based conflicts is also surely related to the low practicality of the stimuli that can induce a tilt-translation ambiguity. It requires sustained high accelerations (e.g., as on aircrafts taking off) which are difficult to reproduce in laboratory conditions except with complex motion systems such as a centrifuge, multi-axis turntables (off-vertical axis rotation) or sledges (large oscillations with high accelerations), and is often highly uncomfortable for participants (24, 29). The latter is particularly critical, as visual and vestibular rehabilitation have been shown to be effective only when they can be administered gradually, since the onset of motion sickness, besides discomforting the patient, jeopardizes training results (30).

The hilltop illusion is a perceptual phenomenon induced by the tilt-translation ambiguity and is caused by repetitive stimulation of the otoliths during linear, low frequency, horizontal oscillations when visual cues are absent (31). Individuals do not perceive the motion as a flat horizontal displacement but as going up and down a small hill, so that an illusion of tilt is created and combined with the horizontal motion sensation. It directly reflects the challenge of perceiving angular motion at low frequency, which leads to an ambiguity on the estimation of the direction of gravity reflected in a partial reinterpretation of the GIA tilt induced by inertial acceleration as tilt with respect to gravity (32). Without visual cues correcting it, this leads to an illusory perception of tilt.

Previous studies attempting to investigate this illusion were challenged by two aspects: the difficulty to induce a low frequency linear acceleration strong enough to elicit a relevant tilt of the GIA and the quantification of the tilt illusion.

The first challenge is intrinsically related to the physics of stimuli supporting the illusion. During horizontal linear oscillations, the tilt of the GIA is the arctangent of the ratio of gravitational and inertial acceleration. This implies that to have a noticeable tilt, the inertial acceleration needs to be proportionally appreciable relative to gravitational acceleration. However, low frequency oscillation with high linear acceleration requires large displacements, which are usually unfeasible for most laboratories. Previous studies addressed this problem by combining results obtained with different motion devices, using a linear sledge (limited in peak acceleration and length) for testing the illusion on frequencies above 0.2 Hz, and centrifuges or rotating chairs (which use centrifugal acceleration), for testing lower frequencies (32). Besides low reproducibility due to limited access to these devices, such an approach has other limitations. A recent study questioned the validity of comparing results obtained with motion paradigms induced by different apparatuses (33). Although the main characteristics of the perceptual responses were the same across paradigms, differences were observed that could be explained with the supplementary sensory input induced by each motion paradigm. Additionally, a virtual reality study showed that a sense of agency over one’s actions as well as personal experiences and expectations can lead to an overestimation of the hill’s slope in the hilltop illusion (34). Similarly, it has also been demonstrated that cognitive signals can suppress the illusion (35). These results are compatible with the statement of Von Baumgarten et al. (31) that “The best illusions were experienced by subjects who were brought blindfolded into the laboratory and did not know on what kind of vehicle they were riding. Great care had to be taken to shield the subject from additional positional cues such as given by light, noise and vibration.” Altogether, this suggests that a proper hilltop illusion requires the subjects not be aware of the (actual and potential) physical motion of the device inducing the illusion.

The second challenge, quantifying the tilt illusion, has been addressed directly by asking participants to report on the experienced motion (35, 36), or indirectly, assessing the direction of perceived vertical. The methodology, however, varies considerably from verbal reports to haptic vertical reported with a joystick (33) or subjective visual vertical, implemented either as a continuous adjustment of a luminous line or a single interval task and corresponding psychometric analysis (32, 33, 37). These differences were considered to relevantly affect the results of the aforementioned studies and, once again, a role of the awareness of the protocol (prediction, expectation) was suggested a possible confounder (35).

The results of the studies demonstrate that the two challenges are strongly connected. The gain of the illusion declines rapidly with increasing frequency, with values below 0.1 at frequencies above 0.1 Hz (32, 37). Slightly higher values are found when using centrifuges rather than devices providing purely linear translations (33), stressing the need to use large devices for exploiting this illusion. Removal of the motion expectation by bringing the participant blindfolded on to the motion simulator, as done by Wertheim et al. (35), led to stronger perceptual responses. However, it still required translations of 1.6 m at 0.16 Hz to obtain a reported tilt sensation in roughly 50% of the participants, a value dropping below 30% with lateral motion of 0.8 m.

Taken together the considerations support the predominant expectation that the hilltop illusion may have little practical use as it is hard to obtain, except with complex devices, and hard to assess, because of its sluggish nature and the weakness of the response.

In contrast, we hypothesize that a practical implementation of the hilltop illusion can be achieved using small amplitudes of linear displacement and low oscillation frequencies, if any cognitive bias on the motion illusion could be neutralized. This approach will allow a stimulus that can be delivered by a wider range of motion devices and is therefore easily reproducible, while being capable of providing a reliable, quantifiable measure of the tilt-translation illusion. At the same time, being based on small amplitudes it will highly be tolerable for participants.

We studied whether it is possible to create a protocol using stimuli in a frequency range where the tilt-translation ambiguity is critical (i.e., 0.1–0.2 Hz) to induce a hilltop illusion that is implicit, practical and effective. *Implicit* here means an illusion that may have been perceived but is not consciously registered. For this we aim to further expand the hypothesis of Wertheim et al. (35), not only preventing expectation on the actual motion, but also limiting any conscious attempt to interpret the sensed motion. *Practical* in the sense that it can be implemented on a standard motion simulator with a limited range of motion (such as a commercial Stewart platform) and that does not cause discomfort to the participants. We decided to test lateral translations at 0.16 Hz, which was reported to be the cut-off frequency for the tilt-translation ambiguity (38, 39). This frequency is also the lowest ever tested on a standard motion platform (without the use of centrifuges) to quantitatively assess the illusion of tilt (35, 36) and would thus demonstrate the practicality of producing this illusion on a small simulator. With *effective*, we mean that the presence of the illusion, although implicit, can be quantified. To this aim we set up to assess the hilltop illusion using both the objective methods previously used in the literature, subjective haptic vertical and subjective visual vertical, and compare the outcomes. Although several studies explored the difference between haptic and visual assessment of verticality (40–42), they were mostly focused on large tilts. In these conditions, the A-effect is known to bias visual assessment (43), and the aforementioned studies mainly consider the discrepancy with respect to this distortive visual effect. In our study, we will only induce small deviations from verticality, where little is known about the performance of visual and haptic vertical. Furthermore, our study focuses on a condition where actual tilt, GIA tilt and perceived tilt are not aligned. As it has been suggested that haptic and visual vertical access different gravity estimates (40, 42) it is particularly important to compare how they perform in this specific situation. This comparison will therefore improve comparability of previous results and help select the optimal method for our protocol.

The resulting protocol is expected to deliver significant practical value. First, as it could be adaptable to most simulators and Stewart platforms, it will favor studies on this illusion. These can support the design of virtual reality systems exploiting motion to reduce sickness or improve navigation (44). In addition, as the illusion will be subtle but measurable, it could be integrated in anti-motion sickness training protocols without excessively challenging participants. The protocol could contribute to better diagnostic and therapeutic approaches for vestibular disorders, which often manifest as spatial disorientation (45). In the last decade, combinations of vestibular stimuli with manipulation of visual cues using VR (46) have been studied for vestibular patients’ rehabilitation programs and motion sickness desensitization programs for lay people. Patients with an over-reliance on visual cues (38) or chronic maladapted self-motion perception (21) clearly benefit from these novel multisensory adaptation paradigms that appear successful overall (45).

Vestibular training is also popular for aircraft pilots and in extreme sports. As a direct application, it may additionally be relevant in the training of astronauts, assessing their degree of adaptation to self-motion perception in space and supporting their re-training to 1 g-level. Indeed, when gravity is absent or altered (i.e., during spaceflight missions), static tilts of the head do not influence otolith output. This leads to a disruption between sensory inputs coming from visual, proprioceptive and vestibular receptors. With such a conflict, it has been theorized that in 0-g, otoliths signals are reinterpreted by central networks into linear movements only (namely the otolith tilt-translation reinterpretation or OTTR) (47, 48). D. M. Merfeld further theorizes in a Rotational Otolith Tilt Translation Reinterpretation (ROTTR) hypothesis that during spaceflight adaptation, the nervous system loses its ability to rely on rotational cues to orient itself to gravity (38). Amidst such disturbances between expected and actual sensory feedback, it is not surprising that exposure to sustained free fall results in Space Motion Sickness (SMS) in over 70% of astronauts (49, 50).

## Methods

### Participants

Data collection was done in 12 healthy participants (6 males, 6 females, average age 29.7 ± 14.6 yo). All participants were free of any known vestibular or neurological disorders.

The protocol was approved by the local ethics committee (Cantonal Ethics committee Zurich, BASEC 2022-01533) and was in accordance with the ethical standards laid down in the 2013 Declaration of Helsinki for research involving human subjects. Before the experiment, participants received explanations on the capability of the motion device and were instructed on the tests to be performed. They agreed to participate and signed an informed consent.

### Experimental setup

The study took place in the Zürich University Hospital (UZH), Zürich, Switzerland. The motion stimulus (physical translations and tilts) was provided with a Stewart platform (hexapod; E Cue 624-1800 motion system, FCS Simulator Systems, Schiphol, Netherlands) with 6 degrees of freedom (3 directions for linear motion and 3 axes for rotational motions). Participants were comfortably seated upright on a seating system mounted on the platform and secured with a four-point safety belt. Padding cushions were placed around legs, hips and shoulders to minimize proprioceptive feedback. The head was secured with a thermoplastic mask, individually molded to each participant to minimize head motion. Acoustic cues were masked by noise-canceling earphones and music. The head was positioned so that the roll rotation axis bisected the interaural line. Video and audio communication allowed monitoring of the participants during the experiment.

### Motion stimulus

**Figure 1:**
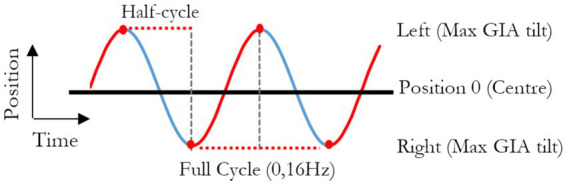
Graphical representation of the position of the platform through time, in reference to the room. A full cycle represents the platform moving from one side of the room to the other, then back to original position and lasts 6.25 s (0.16 Hz).

All experiments were performed in total darkness. The motion stimulus consisted of 71 cycles of sinusoidal sway (interaural translation) with an amplitude of 0.44 m (i.e., 0.88 m total displacement from left to right) and a frequency of 0.16 Hz (Figure 1). During the development of the protocol, a bias toward reporting lateral translation-only displacement became apparent, likely because of motion expectation, mirroring the findings of Creem-Regehr et al. (34). In line with our aim of minimizing cognitive bias, real random roll movements were added to the sequence to foster uncertainty and remove expectations. These rolls consisted of sinusoidal angular rolls at 0.32 Hz with an extent of displacement ranging from −6° to 6°, in steps of 2°, randomly counterbalanced across all cycles. They were also shifted with respect to the linear motion so that the peak of physical tilt occurred at the center of the range of linear displacement, when GIA tilt is 0°, and no physical tilts occurred at the extremes of platform displacements, where GIA tilt peaks. This allowed a decoupling of physical tilt from the assessment of the GIA induced illusion of tilts (i.e., the hilltop illusion). By randomizing the roll angular displacements, we also ensured that any potential effect (or aftereffect, when considering the two extremes) would average to zero over 71 cycles.

Motion of the platform was sampled at 1,000 Hz. A moving average of platform position over 100 ms was calculated to reduce noise, and velocity and acceleration were computed Considering the peak inertial acceleration and gravitational acceleration (*G* = 9.8 m/s^2^), we can calculate the angle *α* of the resulting peak tilt of the GIA vector experienced by the participant:


$$\alpha =\tan^{-1}(\frac{\text{Iner}.\mathrm{Acc}.}{\text{Gravity}})=\tan^{-1}(\frac{0.45}{9.8})=2.63^{{}^{\circ}}$$

### Assessments of outcome variables

Perceived vertical was used to estimate the perception of tilt with respect to gravity, as in previous studies (32, 37). If the perceived vertical deviates to one side with respect to the real vertical, it implies a subjective perception of tilt to the opposite side. Perceived vertical was assessed with two methods, haptic vertical (HV) and subjective visual vertical (SVV), in separate sessions. The order of presentation for the two methods was randomized across participants. Practice trials were provided for the participants to ensure that they were comfortable with the assessment methods, but no feedback was provided.

HV was assessed continuously during the stimulus, using a custom handle (41) secured to the chair for a natural position of the participant’s arm. The participants were instructed to keep the handle aligned with what they perceived as earth vertical. The angle of the handle with respect to the real earth vertical is used as an outcome variable. Data was sampled at 1,000 Hz.

SVV was recorded with single interval task, similar to Pomante (37). The participant indicated whether a briefly displayed luminous line was tilted to the left or right of perceived earth vertical. To display the luminous line at the different SVV test angles, a custom LED bar (31 cm long, 2 mm wide) was fixed on the platform at 150 cm distance from the participant and at eye level so that it always appeared directly in front of the subject. The LEDs were triggered each time the platform reached the extreme translational points and were on for 100 msec for each presented test angle. A two-button controller was strapped in front of the participant to collect responses. Displayed angles were between −5 to 5° with an increment of 1°, and the presentation of angles was randomly distributed to prevent expectations from participants, as suggested by Wichmann and Hill (51). The SVV was collected at the extreme position of the platform only, where the GIA tilt is maximal, and the physical tilt was absent. With this setup, 71 trials were collected at each extreme of the platform, i.e., at each maximum of GIA tilt.

A motion sickness assessment questionnaire (MSAQ) able to describe four dimensions of motion sickness (gastrointestinal, central, peripheral, and sopite-related) and developed by Gianaros et al. (52) was used, and we computed the average score.

### Data analysis

**Figure 2:**
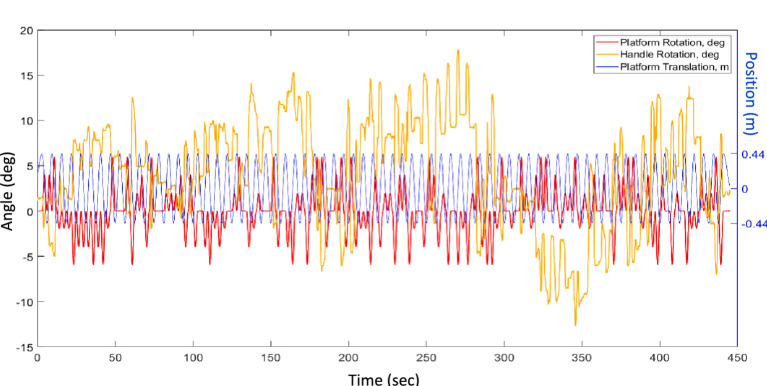
Example of raw data, displaying the repeated translations of the platform (Ty, blue), its random roll angles from −6 to 6° (Rx, red) and the trace of the handle angles performed by a participant (Bar, yellow).

The analysis of the signals was performed in Matlab® R2023a. For the HV, the signal from the handle was processed to extract the perceived tilt due to the hilltop illusion. The physical tilt of the platform (Figure 2, red—added to minimized bias, see “Motion stimulus” section) was subtracted from the tilt reported using the handle (Figure 2, yellow). The resulting signal was detrended and averaged across the 71 cycles of lateral oscillation (Figure 2, blue). This process ensured removal of responses to the randomly counterbalanced physical tilts, as they will average to zero. The result was the average tilt error across all cycles of linear motion.

For the SVV, the data at each extreme of the linear displacements generated two psychometric curves with 71 trials each. The two curves were analyzed separately by fitting a cumulative Gaussian function to extract the parameter *μ* (mean) and *σ* (standard deviation), corresponding, respectively, to the estimated perceived vertical and its uncertainty.

### Statistics

Statistical analysis was performed with GraphPad® Prism, version 9.0.0. All variables were tested for normality using Shapiro–Wilk test and accordingly mean and standard deviation or median and interquartile range were used.

## Results

### Haptic vertical (HV)

**Figure 3:**
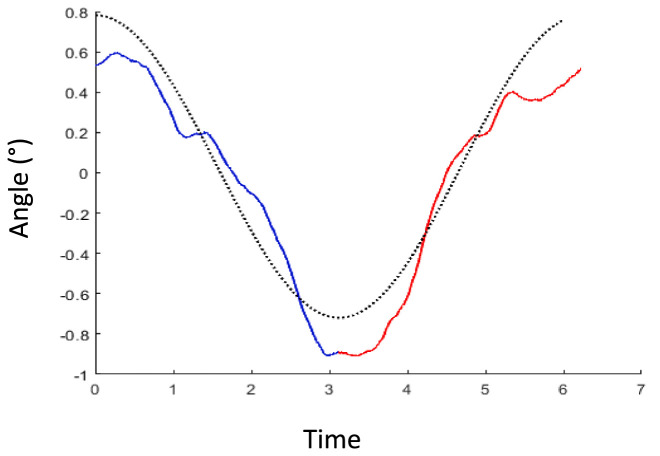
Example of average handle movement for each translation cycle in one participant. The blue trace represents the average of all handle curves during the 71 half-cycles, where the platform travels from left to right. The red curve is the mean trace of handle recordings for all half-cycles going right to left side. The dotted black curve shows a typical one cycle sinusoidal trace with the amplitude of this participant’s data.

**Figure 4:**
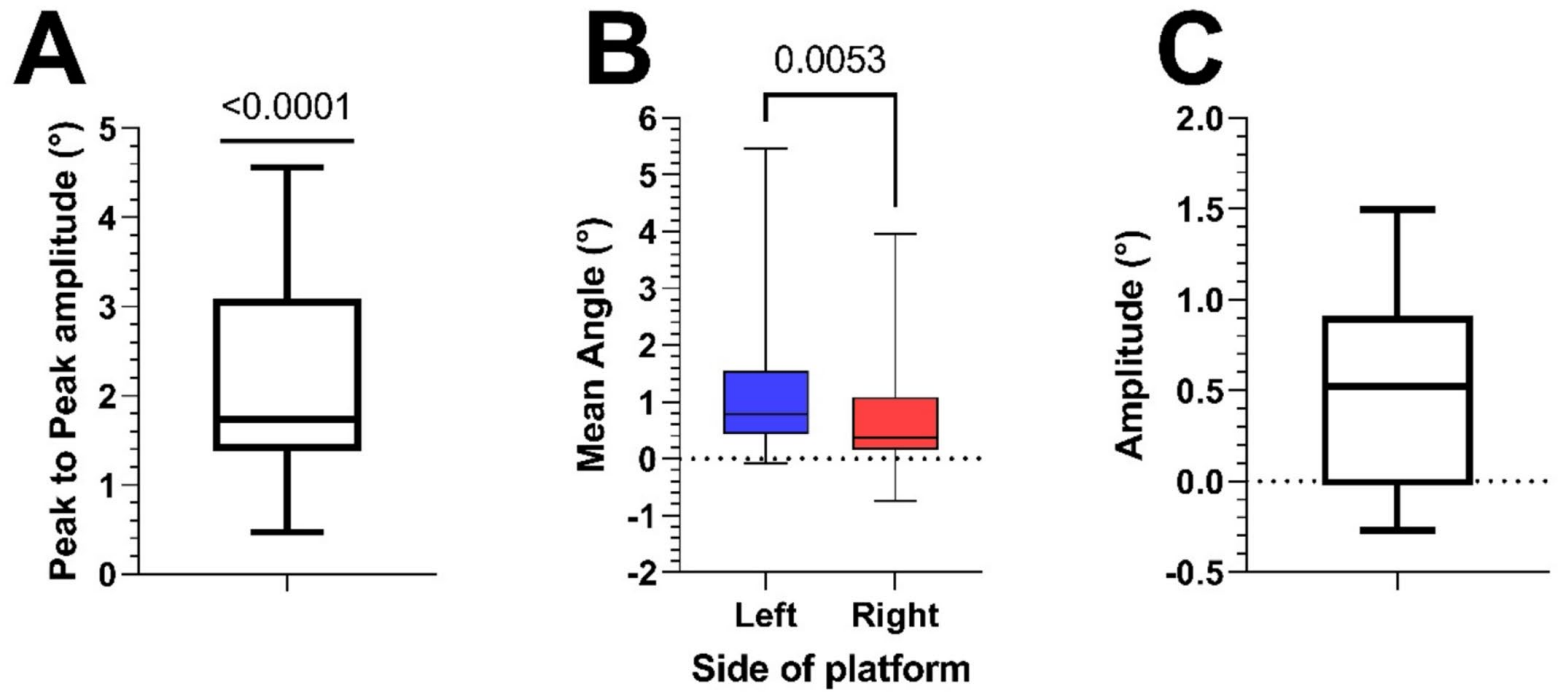
**(A)** Boxplot of peak-to-peak amplitudes of HV traces of all participants (mean = 2.2° ± 1.2). **(B)** Boxplot of the perceived angles at both sides of the platform during the SVV measurement in all participants (mean Left = 1.42° ± 1.79, mean Right = 0.91° ± 1.47). **(C)** Boxplot of the difference (Left minus Right) of the SVV measurements at the two extremes of the platform (0.51° ± 0.57).

Figure 3 shows the average reported tilt over 71 translation cycles in a typical participant. The averaged trace was modulated as sinusoidal deviation from the true vertical, consistent with the expected deviation induced by hilltop illusion, plus an offset. If the participants had no illusion of tilt caused by the translation, this curve would be flat as the random physical tilt averages to zero. To analyze the strength of the illusion, the peak-to-peak difference was extracted by the signal of each participant The values were normally distributed and mean ± standard deviation was 2.2° ± 1.2, significantly larger than 0 (*p* < 0.0001—Figure 4A).

### Subjective visual vertical (SVV)

**Figure 5:**
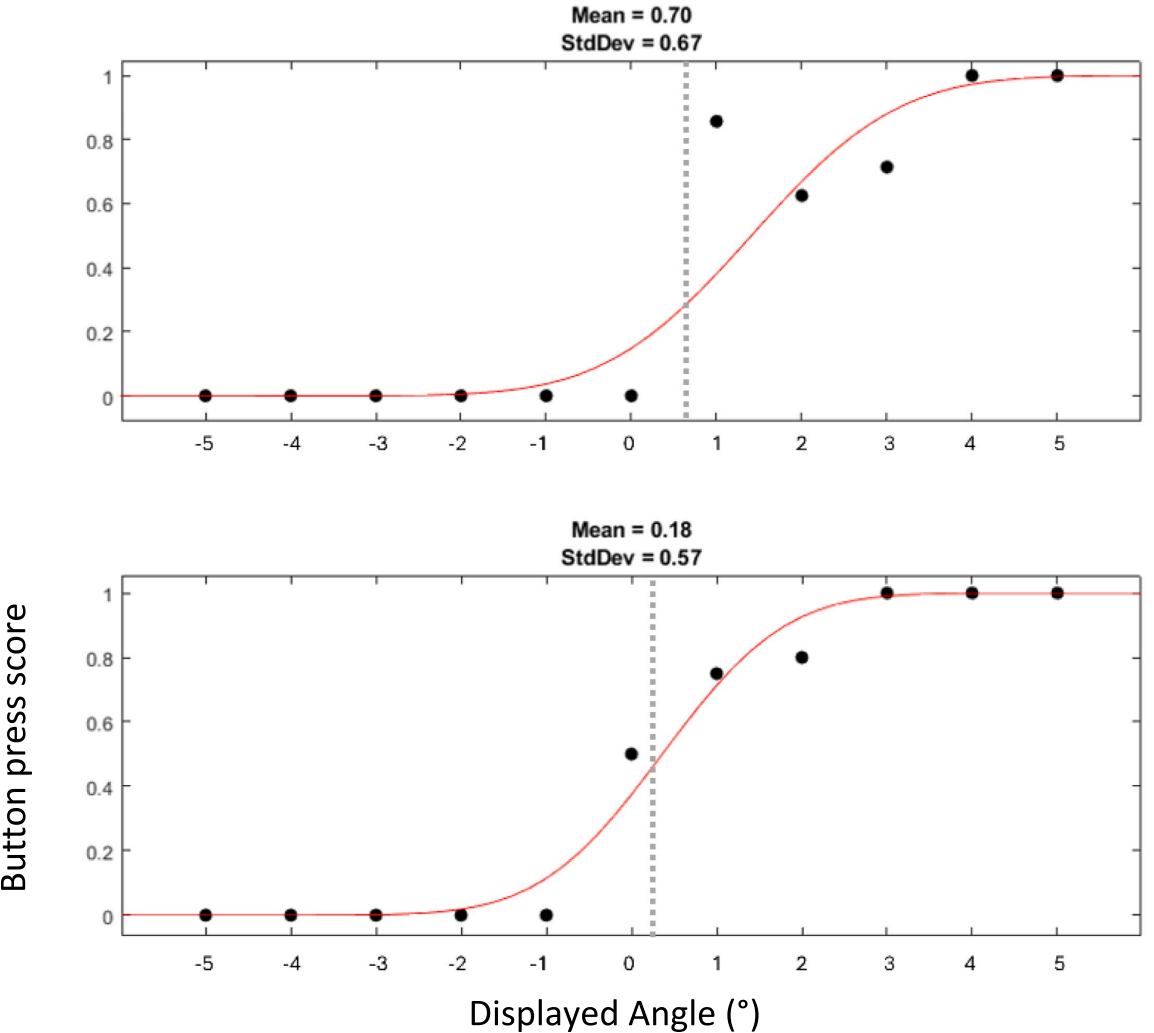
Example of two psychometric curves of SVV collected from one participant. The top curve represents responses when the platform was at the left extreme, and the bottom curve those at the right extreme. The dots displayed with the curves indicate the proportion at which a rightward tilt was endorsed. Thus, a score of 1 or 0 indicates that a rightward or leftward tilt, respectively, was perceived in all trials for the given angle of the luminous line. It is expected that displayed angles closer to 0° lead to more uncertainty in perception and thus to scores closer to 0.5. Dashed gray line represents the mean angle measured with SVV.

Figure 5 shows two psychometric curves of SVV data collected from a typical participant at the leftmost and rightmost extremes of the platform. The Shapiro–Wilk test confirmed that the mid-points of the fitted psychometric functions (SVV) were both normally distributed. Across subjects, the SVV at the left extreme of the platform (1.42° ± 1.79) were significantly larger (paired t-test, *p* = 0.005) than that at the right extreme (0.91° ± 1.47) (Figure 4B). As these values suggest that most subjects showed a rightward bias, we calculated the midpoint of the two SVV of each subject (53). Adding all values measured on the left and right positions of the platform, then dividing them by 2 gave us a mean angle of 1.17°, with a one sample t-test showing it to be significantly different from 0 (*p* = 0.03). To estimate the size of the tilt illusion, we therefore calculated the difference between the values at the two extremes of the platform. The mean difference between the two values within participants was 0.51° ± 0.57 (Figure 4C).

To better compare the two methods’ estimate of the amount of GIA tilt reinterpreted as tilt of the perceived vertical, we calculated it in the form of a gain, i.e., the ratio of the perceived tilt to the actual GIA tilt. We divide the peak-to-peak value of the HV and the difference of the two SVV by two, thus obtaining an estimate of the average unilateral tilt of perceived vertical.


$$\text{Haptic Vertical}\;(\mathrm{HV}):(\frac{\left(\frac{2.2^{{}^{\circ}}}{2}\right)}{2.63^{{}^{\circ}}})*100=41.8\%.$$

$$\text{Subjective Visual Vertical}\;(\mathrm{SVV}):(\frac{\left(\frac{0.51^{{}^{\circ}}}{2}\right)}{2.63^{{}^{\circ}}})*100=9.7\%$$

**Figure 6:**
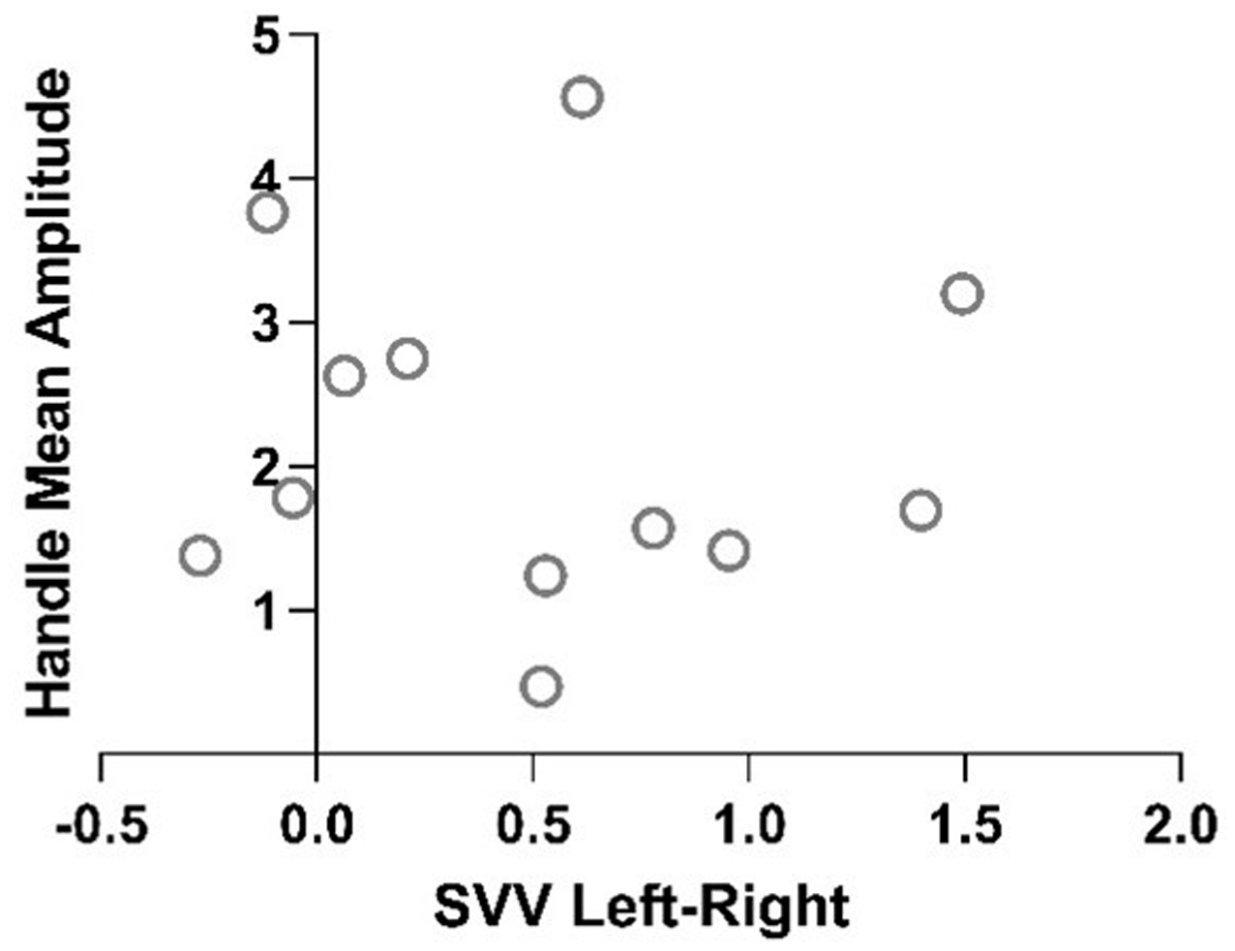
Plot of Pearson R correlation of the two methods of measurements of perceived vertical for each participant.

This results in a 4.3 times larger tilt perception assessed by HV than by SVV. In addition, the two measures did not correlate within subjects with a Pearson R correlation *p* value of 0.46 (Figure 6).

**Figure 7:**
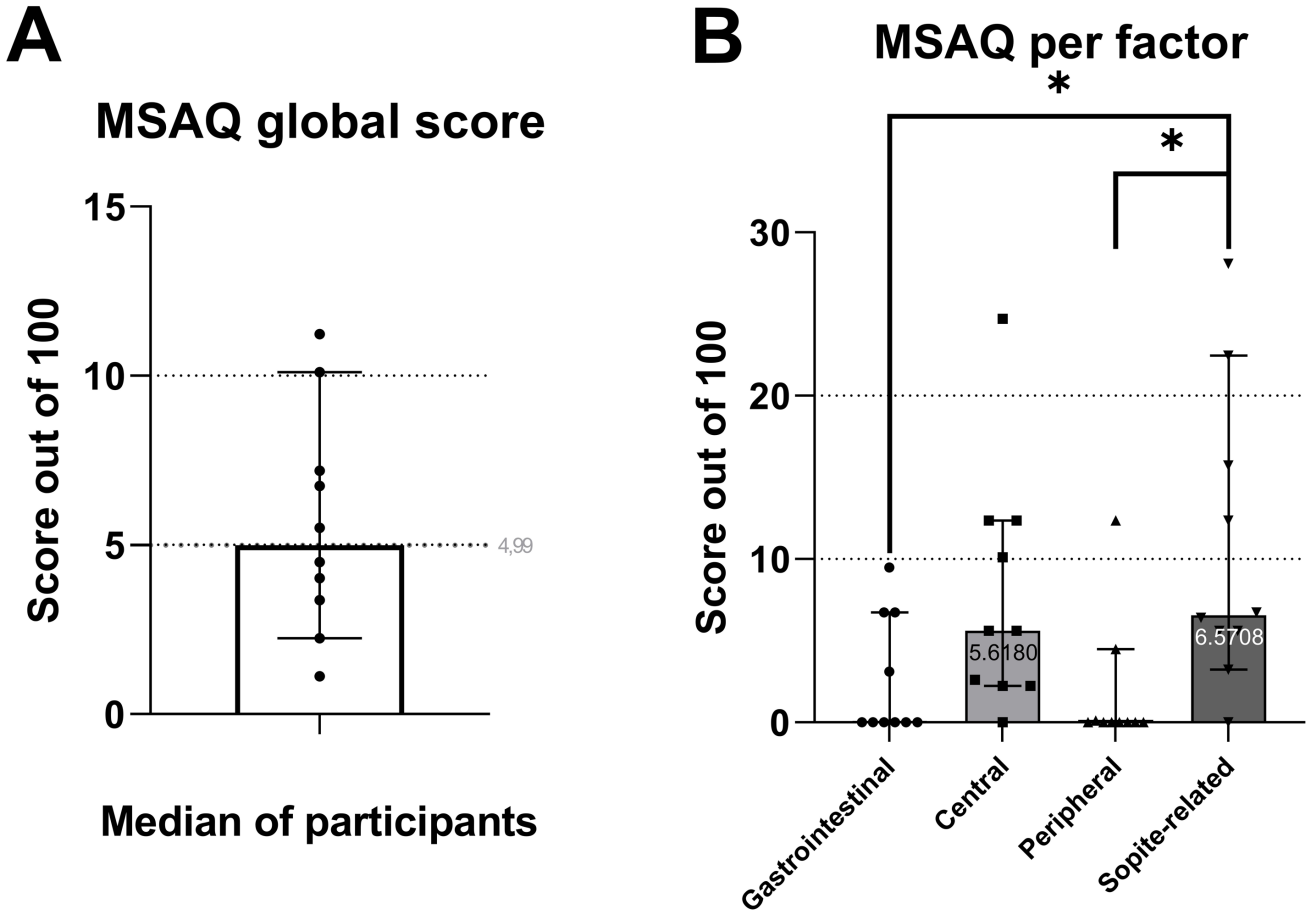
Box plots of MSAQ global score **(A)** and sub-factors **(B)**.

Motion sickness questionnaires showed little to no symptoms with a mean MSAQ (52) global score of 5.6 ± 3.26 out of a maximum of 100 (Figure 7). It can however be noted that with a one-way ANOVA, the mean Sopite-related score (10.63 ± 8.9) is significantly higher than the Gastrointestinal (*p* = 0.04) and Peripheral scores (*p* = 0.02).

## Discussion

A perceptual illusion of tilt was successfully induced by an oscillatory linear motion with a frequency of 0.16 Hz and with a peak acceleration of 0.45 m/s^2^. This acceleration is less than half of those adopted in previous studies using objective assessments. The only study testing a comparable stimulus (0.4 m/s^2^ at 0.159 Hz), collected verbal descriptions of the experienced motion (35). This resulted in only 30% of participants reporting a sensation of tilt, further corroborating the overarching expectation that the hilltop illusion is hard to obtain and assess, except with strong accelerations, requiring large devices.

Our results contradict this expectation. The tilt illusion obtained with our paradigm was quantifiable with two different assessment methods, namely HV and SVV, respectively with gains of 41.8 and 9.7%.

This demonstrates that we developed an assessment protocol that allowed us to induce and quantify the hilltop illusion, i.e., a vestibular induced illusion of roll tilts when only low frequency lateral oscillations are present, despite a small amplitude of lateral acceleration.

The results have a direct practical impact, demonstrating that the hilltop illusion can be realized on most motion platforms. This will support its implementation in further studies as well as the development of diagnostic and rehabilitation protocols based on the tilt-translation ambiguity.

An aspect that may explain the efficacy of our protocol was the effective absence of awareness of the motion profile. The importance of this aspect in inducing the illusion was already demonstrated by Wertheim et al. (35), by preventing the participants from knowing the capability of the motion devices used. In our study, the participants were presented a motion platform capable of performing tilt and translational motion, preventing any bias toward tilt or translation. In addition, we conceived a motion stimulus that effectively masked any awareness of the triggered tilt-translation ambiguity: the combination of the oscillatory translations (0.45 m/s^2^ at 0.16 Hz) with phase shifted and randomly counterbalanced physical tilts. This led to a motion paradigm that provides tilts, whose size (1°–6°) outweighed the illusory tilts induced by the GIA tilts, reducing the possibility that the participant would be aware of the illusion.

Our results therefore corroborate and further stress the importance of the finding of Wertheim et al. that the expectation of the kind of motion plays a key role in the genesis of the illusion. We further developed the concept, hypothesizing that, even in the absence of overt expectations, the awareness of the motion illusion would trigger cognitive processes that may suppress or alter the illusion. The effectiveness of our protocol, capable of identifying an average illusory tilt as small as 0.25° (as measured with the SVV), supports our hypothesis.

We observed a striking difference between the amplitudes of the tilt illusions reported with the two testing methods. Differences between HV and SVV are known from previous studies employing actual tilts (54–57). These differences alone, however, are unlikely to explain the 4-time larger tilt reported with HV compared to SVV. Furthermore, the HV and SVV measures did not correlate, suggesting that different mechanisms may have been in action. In their visual vertical experiment, Pomante et al. (37) observed findings that aligned with predictions of a Bayesian inference model. However, phase shifts and modulation amplitudes differing from theoretical expectations suggested that non-vestibular factors, such as sensory feedback (skin pressure, noise) and cognitive expectations, could play a role in gravity perception. This might thus also be part of the reason why we obtained such a large difference in measured angles between HV and SVV assessment methods, and it should be critically considered. The HV traces of several participants were also affected by drifts, which we removed by detrending. While this might be related to known hysteresis and drifts observed when assessing verticality with methods based on manual adjustments (58–60), participants spontaneously reported more confusion in the HV than the SVV task. In addition, it can be questioned what is being assessed with this method: perception of verticality, perception of GIA tilt forces (61, 62) or dexterity?

On the other hand, even though SVV is now a widely used and standardized test for saccular and utricular functions (63), numerous studies on SVV testing small roll angles identified an overestimation of tilt, a phenomenon named the E-effect (64). One of the proposed interpretations (65) is that the retinal image processing does not fully compensate for the ocular counter roll during static tilt (i.e., the rotation of the eyeball partially compensating head tilts to facilitate stable vision), resulting in a systematic error. A definitive consensus on this hypothesis has not been reached and some results contradict it (66, 67). The potential contribution of ocular counter roll to our SVV results needs to be considered, due to the magnitude of our GIA-tilt and of the SVV results. Indeed, Park et al. (68) reported that for a static head tilt of 2°, ocular counter rolling could show a gain value of up to 0.4. Similar gains (0.26–0.45) have been reported by Fesharaki et al. (69) for tilt angles of 5°. For dynamic linear translations, however, only 2° of eye roll has been observed for 0.2 g of peak lateral acceleration at 0.2 Hz (70), implying a gain of 0.16. Although relatively small, this value is superior to the Hilltop effects we and others (32) observed for the whole tilt illusion. Considering our peak stimulus of 0.046 g would lead to an ocular counter roll of 0.46° per side, which would indicate that the overestimation (E-effect like) is larger than the effect we measured (0.51°/2 = 0.255°). This explanation does not seem to have internal consistency. According to it, a rightward interaural acceleration stimulus induces a rightward counter roll of the eye of 0.46° (compatible with the leftward tilt illusion of the hilltop). If this counter roll is uncompensated, an SVV error of 0.255° implies a perceived vertical deviated to the left of 0.255° (consistent with a rightward head roll, i.e., against the Hilltop illusion and against the motion consistent with ocular counter roll). Partial uncompensated ocular counter roll leads to a less extreme scenario, which may be considered more realistic, and would imply that we largely overestimated the hilltop effect. However, a similar consideration would have to be applied to studies with stronger lateral accelerations (32) where the SVV gain (0.066) will also be largely outweighed by the counter roll gain of 0.16 (70). In similar conditions, participants reported a hilltop illusion (35), implying that the tilt is not significantly smaller to that using the SVV. Furthermore, the presence of an OCR implies that the brain thinks the head is tilted, so it is unlikely that our small effect is being overestimated. Taken together, this suggests that the role of uncompensated ocular counter roll is likely minor in this protocol.

Previous research by Diamond et al. (71) interestingly suggested a higher degree of ocular counter rolling during rightward head tilts. This asymmetry may have relations to the one we observed in the SVV results. Future investigations could further clarify this relationship by quantifying ocular control under the same stimulus paradigm.

The perceived tilt of the SVV is in line with previous studies. Specifically, although no previous study assessing SVV used our stimulation parameters, Glausauer (32) found a gain of 6.2% at 1.33 Hz and 6.6% at 1.0 Hz using a continuous SVV assessment method, values lower but not too different from the 9.7% found here. The GIA tilt amplitude was however, respectively, 3.2 and 1.9 time larger in Glasauer’s study reaching 8.5° at 1.33 Hz and 5° at 1.00 Hz, while it was of 2.63° in this study. As already shown by Pomante et al. (37), the choice of estimating the SVV using a psychometric approach on data from a single interval task produces a higher sensitivity to small deviations compared to the continuous assessment method. This made it possible to reliably assess a peak-to-peak modulation of the SVV of 0.51° ± 0.57, a value comparable to the one reported by Pomante et al. (0.62°). They, however, tested a frequency twice as large as in the current study and lateral accelerations were almost 4 times larger (0.33 Hz and 1.75 m/s^2^) resulting in a gain as low as 2%. In contrast to both studies, we did not collect data at multiple points along the sinusoidal modulation of GIA, but only at the extremes of the lateral motion at the time of peak GIA tilt. This did not allow us to get a sinusoidal fit of the tilt. As a phase shift in the tilt illusion with respect to the lateral motion is expected (72–75), our estimates of the amplitude may be underestimated as we failed to record the peak values. On the other hand, both studies discussed above found that the observed phase shifts were lower than model predictions. Considering the estimate of Glasauer (15.6° phase shift at 1.33 Hz und 28.4° at 1.0 Hz) our error would be less than 10%, acceptable given the advantage in practicality in our protocol. Accordingly, visual inspection did not reveal a relevant phase shift in our HV.

Our protocol was well tolerated. Motion sickness was close to absent, suggesting that this protocol inducing a subtle hilltop illusion with no significant discomfort is a good candidate as a basis for providing adaptation cues for training (e.g., for progressive training pilots or astronauts to conflict) or rehabilitation.

The efficacy and reliability of our protocol corroborates the idea that a diagnostic, rehabilitation or training protocol exploiting the ambiguity on the estimated direction of gravity occurring at low frequency lateral oscillation can be implemented on practically any motion simulator. A power analysis based on our data suggests that a significant difference can be obtained for a one-tailed paired t-test with as little as 10 participants, a value normally expected in any research study on the topic. It remains critical to ensure that the participants are not aware of the stimuli and to implement an assessment method with the necessary sensitivity to small tilt angles.

## Data Availability

The raw data supporting the conclusions of this article will be made available by the authors without undue reservation.
